# For which cancers might patients benefit most from expedited symptomatic diagnosis? Construction of a ranking order by a modified Delphi technique

**DOI:** 10.1186/s12885-015-1865-x

**Published:** 2015-10-30

**Authors:** Willie Hamilton, Sally Stapley, Christine Campbell, Georgios Lyratzopoulos, Greg Rubin, Richard D. Neal

**Affiliations:** 1 University of Exeter, College House, St Luke’s Campus, Exeter, EX2 4TE UK; 2 Centre for Population Health Sciences, The University of Edinburgh, Medical Quad, Teviot Place, Edinburgh, EH8 9AG UK; 3 Cambridge Centre for Health Services Research, Department of Public Health and Primary Care, University of Cambridge, Cambridge, CB2 0SR UK; 4 Department of Epidemiology and Public Health, University College London, 1-19 Torrington Place, London, WC1E 7HB UK; 5 School of Medicine, Pharmacy and Health, University of Durham, Wolfson Research Institute, Queen’s Campus, Stockton on Tees, TS17 6BH UK; 6 North Wales Centre for Primary Care Research, Bangor University, Gwenfro Unit 5, WrexhamTechnology Park, LL13 7YP UK

**Keywords:** Cancer, Diagnosis, Primary health care, Modified Delphi technique

## Abstract

**Background:**

This study aimed to answer the question ‘for which cancers, in a symptomatic patient, does expediting the diagnosis provide an improvement in mortality and/or morbidity?’

**Methods:**

An initial ranking was constructed from previous work identifying ‘avoidable deaths’ for 21 common cancers in the UK. In a two-round modified Delphi exercise, 22 experts, all experienced across multiple cancers, used an evidence pack summarising recent relevant publications and their own experience to adjust this ranking. Participants also answered on a Likert scale whether they anticipated mortality or morbidity benefits for each cancer from expedited diagnosis.

**Results:**

Substantial changes in ranking occurred in the Delphi exercise. Finally, expedited diagnosis was judged to provide the greatest mortality benefit in breast cancer, uterine cancer and melanoma, and least in brain and pancreatic cancers. Three cancers, prostate, brain and pancreas, attracted a median answer of ‘disagree’ to whether they expected mortality benefits from expedited diagnosis of symptomatic cancer.

**Conclusions:**

Our results can guide future research, with emphasis given to studying interventions to improve symptomatic diagnosis of those cancers ranked highly. In contrast, research efforts for cancers with the lowest rankings could be re-directed towards alternative avenues more likely to yield benefit, such as screening or treatment.

**Electronic supplementary material:**

The online version of this article (doi:10.1186/s12885-015-1865-x) contains supplementary material, which is available to authorized users.

## Background

It is widely believed that the sooner a cancer is diagnosed, the better the outcomes for that patient will be [1]. There is strong evidence from screening trials in some cancers that this is true for the asymptomatic patient. However, once the patient has developed symptoms, the evidence for mortality benefit from expediting the diagnosis is much weaker, and relies on observational data [2–4]. Even so, it is still considered axiomatic that avoiding delay in symptomatic cancer diagnosis is beneficial, in reducing mortality and morbidity, providing symptom relief and possible improvements in psychological well-being. This view underpins the many interventions in recent years aimed at improving survival rates from cancer, especially in those countries with a relatively poor cancer performance, such as the United Kingdom (UK) and Denmark [5].

Patients certainly value early diagnosis of cancer [6]. However, not all cancers behave in the same way and there are many different types. Survival differs greatly across the common cancers; so does ease of diagnosis –shown by the number of primary care consultations pre-diagnosis [7], times to diagnosis [8], and the proportion having an emergency presentation [9]. In some poor-prognosis cancers, there is an element of nihilism with cure considered unlikely whatever the time to diagnosis (though relief of symptoms and prolongation of life remain important) [10]. Such nihilism may pervade the research agenda; for instance, lung cancer has consistently attracted much lower research funding than the other three common cancer sites [11].

If expediting symptomatic diagnosis is of greater (or lesser) value across different cancer sites, then it would help the research community to know which cancer sites have most (or least) to gain. Those with most to gain might preferentially receive research funding targeted on early cancer diagnosis. In contrast, research effort in cancers with less to gain would focus more on prevention, development of effective screening, or treatment.

This study aimed to answer the question ‘for which cancers, in a symptomatic patient, does expediting the diagnosis provide an improvement in mortality and/or morbidity?’ In the absence of trial evidence, alternative methods had to be employed. Thus, we used a Delphi exercise, combining the available evidence with the pooled responses of experts – an approach which has not been tried before to answer this question.

## Methods

### Mortality benefits

#### Phase 1 – Construction of the initial ranking

Phase 1 had two stages. The first used data from a study of international differences in cancer survival published by Abdel-Rahman *et al*. [12] This paper reported for each of the 22 commonest cancers the percentage of additional (deemed ‘avoidable’) UK deaths within 5 years of diagnosis in patients aged 15–99, derived from the highest survival estimates for 13 other European countries, in 1995–1999. There is a widely held view that these international differences represented to a considerable extent *diagnostic* delays [1], though treatment differences, differences in organisation of health care and registration processes will have contributed. By extension, the ranking order from this paper would broadly reflect those cancers where there is the most or least to gain from expedited diagnosis. This created Column A_1_ in Table 1.

**Table 1:** Derivation of the figures for Delphi Round 1

Cancer Site	Column A_1_	Column S_half_	Column A_2_
Breast	26.8	11.2	38.0
Uterus	25	10.2	35.2
Kidney	27.7	7.1	34.8
Prostate	24	10.7	34.7
Melanoma	22	11.5	33.5
Hodgkin disease	20.3	10.9	31.2
Cervix	17	8.8	25.8
Larynx	16.6	8.8	25.4
Colon	12.3	7.2	19.5
Ovary	13.5	5.6	19.1
Non-Hodgkin lymphoma	10.1	8.4	18.5
Rectum	10.2	7.4	17.6
Multiple myeloma	11.7	4.9	16.6
Bladder	8	7.1	15.1
Testis	1.4	12.8	14.2
Leukaemia	8.1	5.8	13.9
Stomach	11.6	2.2	13.8
Brain	9	2.0	11.0
Lung	7.2	1.2	8.4
Oesophagus	4.5	1.7	6.2
Pancreas	2.5	0.5	3.0

The second stage adjusted Column A_1_ to accommodate cancers with high survival, in the belief that the Abdel-Rahman paper would fail to capture fully any survival benefit from expedited diagnosis in this group. For example, testicular cancer has very high survival, and international survival differences are small. However, this could be because the disease is extremely chemo-sensitive (which it is) or because it is a cancer which is diagnosed quickly, or occurs in younger patients – or more than one of these. Therefore, high survival may mask any advantages from rapid diagnosis. For each cancer, UK 5-year survival was identified from the Office for National Statistics publication for England, relating to 2005–9 [13]. For each cancer site, the percentage surviving 5 years was added to the figures in Column A_1_, giving a half weighting to the survival figures (shown in column S_half_). This process led to the removal of oral cavity cancers from Column A_2_, as survival statistics separated oral cancers into subgroups; we could identify no composite figure.

#### Phase 2: the Delphi survey – round 1

We followed best practice in consensus methods for this [14]. We invited 189 participants to a modified Delphi exercise by e-mail, with a reminder to non-responders after three weeks: 22 replied and all participated in both rounds of the Delphi, lasting from March to May, 2014. They were chosen for their general expertise in the cancer field and included general practitioners, public health professionals, academic researchers (targeting individuals in these groups with specific professional responsibilities for cancer) and clinicians linked to cancer networks. We specifically excluded cancer specialists with single site expertise on methodological grounds [15]. The initial invitation list was generated by the steering group; if an invitee recommended a colleague suitably qualified to respond we extended the invitation. The invitation included an explanatory letter, a resource pack and an Excel table for replies. The letter and resource pack described the research question,

‘For this particular cancer, in a patient who has symptoms from the cancer, does expediting the diagnosis provide an improvement in mortality?’

The resource pack was derived from a systematic review first carried out in 2010 [16], and updated in December 2013/January 2014, though published after this study completed [17]. The aim of the review was to determine the impact of different patient, primary care and secondary care intervals (and combinations of these) on stage at diagnosis and/or survival. 121 papers were included. Each was assigned a category based on the study design (see Additional file 1) though most were observational studies of individual patients (category 3). A sample from the resource pack is shown in Additional file 2 (full pack available from authors).

Delphi participants were allocated a ‘purse’ of 40 points to be used to adjust the position of cancers in Column A_2_ [18]. Positive or negative points could be used - both counting equally to the total spend. All 40 points had to be used, with +/−10 being the maximum for a single cancer site. Delphi participants were asked to use their personal experience, plus the evidence in the resource pack, to make these adjustments. We did not request details of how much participants used the resource pack, and how much they relied on personal experience (though most round 1 replies from participants included comments upon the lengthy time involved in making their judgements, suggesting the resource pack was used considerably).

#### Weighting of phase 2, round 1 of the Delphi exercise

We weighted the responses to ensure that Round 1 of the Delphi exercise achieved an overall influence of 33 % for the whole study. Column A_2_ – the initial ranking sent to Delphi participants – totalled 436 points, with a standard deviation of 10.5. The weighting within Phase 2 aimed to give 2/3^rd^ of 436 points to Round 1, i.e. 291 points, to achieve the overall influence of 33 %. The process for calculating the weighting entailed: a) calculation of the mean change in points allocated by Delphi participants to each cancer site (this could be a positive or negative number); b) negative mean changes were temporarily made positive in order to avoid any ‘cancelling out’ effect, and the total of the 21 mean values was calculated to give the overall magnitude of change; c) 291 was divided by this total magnitude of change, to give a conversion factor (8.61); d) originally negative mean changes were restored to negative (reversing stage b); e) individual mean changes for each cancer were multiplied by the conversion factor, and then added to the original points in Column A_2_ to give Column B.

To estimate how much influence the Delphi participants actually had, the adjusted changes can be expressed as multiples of the standard deviation of the values in Column A_2_. The greatest adjusted change would have been if every participant had given one particular site the maximum allowed 10 points. Once multiplied by the conversion factor of 8.61, the greatest adjusted change would be 86 points, or 8.2 standard deviations. Thus any cancer could have been promoted from the bottom of the ranking to the top, or *vice versa*.

#### Likert rating of mortality and morbidity statements

Delphi participants also rated statements relating to mortality and morbidity benefits for each cancer site. These were: ‘Expedited cancer diagnosis brings mortality benefits in this cancer site’ and ‘Expedited cancer diagnosis brings morbidity benefits such as improved treatment options and psychological benefit in this cancer site.’ Treatment options were described further to include pain or other symptom relief. Responses were requested on a 5-point Likert scale: Strongly disagree; Disagree; Undecided; Agree; Strongly Agree. For analysis, we did not assume the responses were equally spaced; instead we report the median response (modal responses differed in only 7 of the 42 answers, always to an adjacent response).

#### Phase 2: the Delphi survey – round 2

All 22 responders to Round 1 of Phase 2 were invited to participate in Round 2. The *pre hoc* principle was that Round 2 should have a total influence on the whole process of 17 %. Each participant was allocated a ‘purse’ of seven points which could be used positively or negatively to one or more cancer sites. Unlike Round 1, participants were not obliged to ‘spend’ all – or any – of their points if they were happy with Column B. Column C was constructed from Column B, plus the mean change in allocated points in Round 2.

To test whether the survival adjustment in the second stage of Phase 1 was reversed in the Delphi process, we calculated the correlation between the change from Columns A_1_ to A_2,_ and the change from A_2_ to Column C (the entire Delphi process). Analyses used Stata, version 13 [19].

No ethical committee review was required for this study, as is the norm with Delphi studies and with research involving staff; participants were deemed to have consented by returning the questionnaire.

## Results

**Fig. 1:**
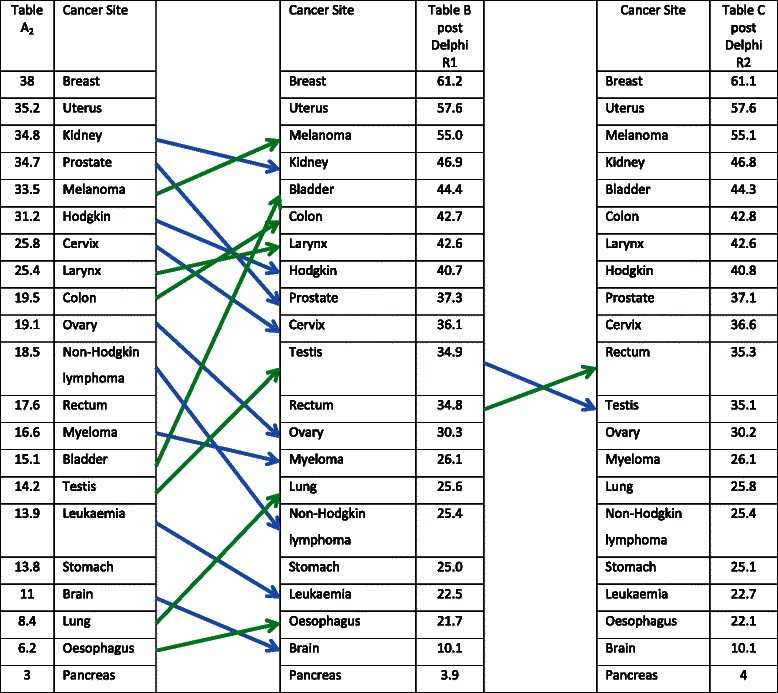
the initial ranking (Column A_2_), plus results of the two Delphi rounds (Columns B and C)

**Figure 2:**
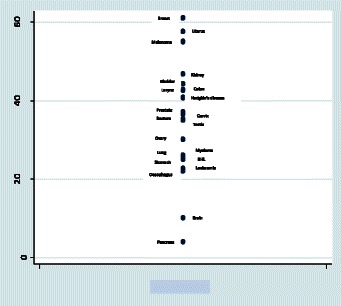
Relative positions for 21 cancer sites in terms of potential benefit to be had from expedited symptomatic cancer diagnosis

Figure 1 shows the results of Phase 1 of the project, which created Column A_2_, plus the changes made in the Delphi process, Columns B and C. In total, 160 UK and 29 international experts were invited to participate in the Delphi process. Of these 189, the email was undeliverable in 12 participants, nine declined, (five being too busy and four considering their expertise insufficient); there were 146 non-responders. Twenty-two completed returns (21 from the UK and one from Denmark) were received: ten academic researchers in cancer diagnosis, six GPs, four clinicians with cancer network responsibilities and two public health professionals with a specific cancer remit in their posts: all 22 also participated in Round 2. Seven allocated no points in the second round, stating Column B was correct. The correlation coefficient between change from Column A_1_ to Column A_2_ (Phase 1) and the change from Column A_2_ to Column C (i.e. Phase 2, the Delphi process) was +0.43 (p = 0.05), suggesting that the survival adjustment in Column A_2_ was helpful. The final relative positions of the 21 cancer sites after the Delphi process are shown in Figure 2. The Likert responses are shown in Table 2.

**Table 2:** Median Likert responses of statements that expedited symptomatic cancer diagnosis brings mortality or morbidity benefits

	Expedited symptomatic cancer diagnosis brings mortality benefits	Expedited symptomatic cancer diagnosis brings morbidity benefits
Strongly agree		
Agree	Bladder, breast, cervix, colon, larynx, lung, melanoma, ovary, rectum, testis, uterus	Bladder, brain, colon, kidney, larynx, leukaemia, lung, myeloma, oesophagus, ovary, stomach
Undecided	Hodgkin’s disease, kidney, leukaemia, myeloma, non-Hodgkin’s lymphoma, oesophagus, ovary, stomach	Breast, cervix, Hodgkin’s disease, melanoma, non-Hodgkin’s lymphoma, pancreas, prostate, rectum, testis, uterus
Disagree	Brain, pancreas, prostate	
Strongly disagree		

Note: the median response for ovary and mortality was between ‘agree’ and ‘undecided’ – thus we have placed it in both cells.

## Discussion

### Summary of the main findings

This is the first study to try to rank cancers by potential mortality benefit from expedited symptomatic diagnosis. The final ranking, although not definitive, suggests that cancers with the greatest potential benefit are breast, uterus and melanoma; in contrast, pancreas and brain were considered to have the least potential benefit. The Likert responses for whether mortality gains were to be expected from expediting symptomatic diagnosis were concordant with the final rankings. Perceived morbidity benefits were largely unrelated to the mortality ranking for cancer sites.

### Strengths and limitations

This study had two distinct phases: construction of the initial ranking and the Delphi process. Both have methodological limitations. The initial Column A_1_ was predicated upon the view that European cancer survival differences reflected differences in timeliness of diagnosis in part at least. We used the best information available, and considered it preferable to provide an initial ranking rather than offering Delphi participants a starting point of all cancers being equal – while accepting the possibility of an ‘anchoring’ effect [20]. This effect occurs when people are given a specific value for a quantity, before measuring (or in our case, adjusting) the quantity itself. The Abdel-Rahman paper uses data which is now close to twenty years old; although improvements in diagnosis and treatment may have changed the ranking somewhat, the purpose of the initial ranking was to provide a reasonable starting point based – in part at least – on diagnostic differences. More recent data – had it been available – would have reflected the improvements in UK cancer diagnostic pathways in recent years, making international variation less helpful in showing up any effect from improved diagnostics.

The adjustment for survival was also debatable; the rationale was that patients with cancers with high cure rates could still benefit from expedited diagnosis. One internal finding suggests that this process was valuable: the positive correlation between the change in scores introduced by the survival adjustment and the change in scores from the Delphi exercise. In short, the Delphi exercise moved in the same direction as the survival adjustment, rather than reversing it. Furthermore, there was ample latitude in the Delphi exercise to change any misplaced cancer. A cancer ranked last could have become first, and *vice versa,* though no cancer changed ranking as dramatically as this. Other choices in Phase 1, including different weightings, would have generated different starting points for the Delphi participants; they might not have led to important differences in the final outputs. One unfortunate effect of the survival adjustment was that oral cancer was lost to the Delphi process, as we could not find appropriate survival figures. The initial position of oral cancer was mid-ranking, between rectal cancer and non-Hodgkin lymphoma. We cannot know what its final position would have been had it entered the Delphi exercise.

The Delphi exercise had two rounds. A feature of the Delphi process is reiteration, with structured feedback and subsequent rounds helping to develop consensus. The number of rounds is a balance between maintaining recruitment and allowing change. We saw little alteration in Round 2 – with nearly a third of the panel suggesting no change at all was needed – and hence the value of a third round would probably have been limited. Our number of Delphi participants was within the usual range [21]; of 189 invited, 22 took part, all of whom completed both rounds. We do not know why most of the non-responders declined. The evidence pack was large, and many participants indicated that the exercise required both considerable time and intellectual engagement. We cannot know if a different panel would have yielded different results. Deliberately, we excluded patients and surgical specialists from the Delphi process: both groups largely have expertise in a single cancer site (or grouped sites, such as urological cancers). We believed that the Delphi participants should be able to comment across a wide range of cancers to allow them to adjust the rankings appropriately. A future exercise could extend to a much wider community, including patients, the public and specialists.

The findings from this study would change if major improvements in treatment for a particular cancer were available. For example, were a new drug for pancreatic cancer to be developed, then expedited symptomatic diagnosis of pancreatic cancer would presumably increase in importance. Therefore, our rankings are of value only for the present time. They also only pertain to the 21 cancers we included. Omission of other cancers from the ranking does not mean no benefit is to be expected, just that any such benefit is unreported.

### Interpretation

This is the first exercise of this nature that we know of. Thus we cannot directly compare it with other published studies. The research question offered to Delphi participants made no reference to division of the pre-diagnostic period into patient intervals (before entry into healthcare) and healthcare provider/system intervals [22]. Thus the results may pertain to both, and hence have possible relevance to either awareness campaigns or the design of diagnostic services.

The findings on the potential morbidity benefit were unexpected. Although no cancer sites attracted a median response of ‘disagree’ or ‘strongly disagree,’ ten had a median response of ‘undecided’. This was despite the emphasis given to the possibility of palliation and symptom relief. Three of these cancer sites – breast, melanoma and prostate – have a sizeable literature relating to over-diagnosis, though this mostly relates to screen-detected cancer. It is plausible that, for these sites, Delphi participants believed that the morbidity benefits were outweighed by harms, though it is hard to see how this could extend to the other seven cancers.

Three cancers – breast, uterus and melanoma – were ranked as being most likely to result in improved mortality outcomes through expedited symptomatic diagnosis, and there was a moderate gap between these cancer sites and the next group. In the UK, diagnostic services for breast cancer are highly streamlined, such that in the English National Health Service women with almost any breast complaint (other than pain) are assessed rapidly for the possibility of cancer. This facility – which is the most comprehensive of all the various cancer diagnostic services – is consonant with our findings. We cannot know if the presence of such a scheme influenced Delphi participants, who could reasonably have inferred from the existence of such a service that it was particularly warranted. The generally accepted benefits of breast cancer screening may have encouraged Delphi participants to consider breast to be particularly worthy of expedited diagnosis [23], even though the screening population is asymptomatic, and were not included in this study.

Uterine cancer has a similarity to breast cancer, in that a single presentation, post-menopausal bleeding, dominates [24]. Services for investigation of post-menopausal bleeding are also well-structured, and timely investigation is the norm. In contrast to breast cancer or melanoma, uterine cancer has not been considered for awareness campaigns encouraging women with post-menopausal bleeding to consult their doctors. Our results suggest this should be considered.

Melanoma also has a characteristic symptom, a pigmented lesion. Times to diagnosis are generally short, emergency admissions are rare, and few patients report seeing their doctor three or more times before diagnosis [7–9]. Some concerns about ‘over-diagnosis’ have been raised for melanoma [25], based on a stable number of deaths in the face of rising numbers of new diagnoses. An alternative interpretation is that good services for diagnosis of melanoma have led to improved survival, despite an increased incidence.

Sixteen cancers are ranked in the middle of the ranking, though for only one of them, prostate, did the Delphi respondents give a median Likert answer of ‘disagree’ to the statement that expedited symptomatic cancer diagnosis brought mortality benefits. Five cancers within this large grouping had large changes in the Delphi exercise: bladder and lung were promoted, and ovary, prostate and non-Hodgkin’s lymphoma fell. The promotion of bladder may represent the clear survival advantage of early bladder cancer diagnosis when compared with advanced disease. The promotion of lung cancer refutes some of the nihilism attaching to this subject; in the UK at the time of the Delphi exercise there was considerable public publicity of lung cancer symptoms, as part of the Be Clear On Cancer campaign. Furthermore, lung cancer resection rates are rising, and newer treatments, tailored to specific cancer genotypes, have entered mainstream practice. This circumstantial evidence may have encouraged Delphi participants to believe that expedited symptomatic diagnosis has benefits – for some lung cancer patients. Ovary is similar to bladder cancer in its sharp survival difference between early and advanced disease, though, in this cancer, advanced disease is the norm. Diagnostic testing has become much easier, with the widespread use of Ca125 [26]. It is hard to explain why non-Hodgkin’s lymphoma fell. Long-term results from a trial of surgery in prostate cancer have yielded small benefits, though with additional morbidity [27]. This is not the same as demonstrating benefits from expedited diagnosis. Furthermore, prostate screening trials have disappointed, reporting no, or very small benefits. This leaves a confusing picture, of small benefits at best – commensurate with its final place in the ranking.

Two cancers were deemed to be associated with the smallest gains from expedited diagnosis throughout the whole exercise: brain and pancreas. Both have very poor prognoses, and this may have been uppermost in Delphi participants’ minds [28]. Although expediting symptomatic diagnosis in these cancers may have less effect in improving survival compared with other cancers, it does not mean there is no value in trying to do so. The primary message is that we have to be realistic about what it is possible to achieve. A clear implication can be drawn: that research efforts should concentrate on other aspects, such as prevention, development of novel screening tools, including new biomarkers, and new targeted therapeutic agents. Quite rightly, these cancers with poor outcomes should receive preferential attention and funding, but that this should focus on areas most likely to offer tangible benefits.

## Conclusion

This study has produced an initial ranking for the mortality benefit available from expedited symptomatic diagnosis of cancer. It can guide future research directions, to ensure they have the maximum chance of making an impact. It should not be used as an excuse to downgrade research on cancer sites near the bottom of the ranking – but as a pointer towards the optimum research direction.

## Additional files

Additional file 1: **Evidence base.** (DOCX 18 kb)
Additional file 2: **Sample from resource pack.** (DOCX 19 kb)
