# Supplementary material for: For which cancers might patients benefit most from expedited symptomatic diagnosis? Construction of a ranking order by a modified Delphi technique
Source: BMC Cancer. 2015 Oct 30;15:820. doi: 10.1186/s12885-015-1865-x (PMC4627396; doi:10.1186/s12885-015-1865-x)
Supplement: Additional file 1: — Evidence base. (DOCX 18 kb) [file 12885_2015_1865_MOESM1_ESM.docx]

**Additional file 1**

Evidence base

| Category | Population | Intervention | Outcome measure | Study type |
| --- | --- | --- | --- | --- |
| 1 | **Individual symptomatic patients** | Expediting diagnosis (operating in community or healthcare system) | Mortality or survival | RCT |
| 2 | **Individual symptomatic patients** | Expediting diagnosis (operating in community or healthcare system) | Stage shift | RCT / case control study |
| 3 | **Individual symptomatic patients** | None, other than healthcare system delays, or changes in healthcare delivery | Change in stage shift with time / Survival | Observational |
| 4 | **Individual symptomatic patients, presenting as emergencies** | None | Increased mortality with emergencies, above that expected from staging | Observational |
| 5 | **National/regional populations** | Increased diagnostics | Mortality | Observational |
| 6 | **International** | Increased diagnostics/treatment | Mortality | Observational |
| 7 | **Individual symptomatic patients** | None | Improved mortality with shorter diagnostic intervals | Observational studies of diagnostic intervals |
