# Supplementary material for: For which cancers might patients benefit most from expedited symptomatic diagnosis? Construction of a ranking order by a modified Delphi technique
Source: BMC Cancer. 2015 Oct 30;15:820. doi: 10.1186/s12885-015-1865-x (PMC4627396; doi:10.1186/s12885-015-1865-x)
Supplement: Additional file 2: — Sample from resource pack. (DOCX 19 kb) [file 12885_2015_1865_MOESM2_ESM.docx]

**Additional file 2**

Sample from resource pack

| **329** | **Torring** | | **Denmark** | | **2012** | | **Journal of Clinical Epidemiology** | | | **3** |
| --- | --- | --- | --- | --- | --- | --- | --- | --- | --- | --- |
| *Diagnostic interval and mortality in colorectal cancer: U-shaped association demonstrated for three different datasets*  **Objective:** To test the theory of a U-shaped association between time from the first presentation of symptoms in primary care to the diagnosis (the diagnostic interval) and mortality after diagnosis of colorectal cancer (CRC).  **Study Design and Setting:** Three population-based studies in Denmark and the United Kingdom using data from general practitioner’s questionnaires, interviewer-administered patient questionnaires, and primary care records, respectively.  **Results:** Despite variations in the potential selection and information bias when using different methods of identifying the date of first presentation, the association between the length of the diagnostic interval and 5-year mortality rate after the diagnosis of CRC was the same for all three types of data: displaying a U-shaped association with decreasing and subsequently increasing mortality with longer diagnostic intervals.  **Conclusion:** Unknown confounding and in particular confounding by indication is likely to explain the counterintuitive findings of higher mortality among patients with very short diagnostic intervals, but cannot explain the increasing mortality with longer diagnostic intervals. The results support the theory that longer diagnostic intervals cause higher mortality in patients with CRC. | | | | | | | | | | |
| **330** | **Torring** | | **Denmark** | | **2011** | | **British Journal of Cancer** | | | **3** |
| *Time to diagnosis and mortality in colorectal cancer: a cohort study in primary care*  **BACKGROUND:** The relationship between the diagnostic interval and mortality from colorectal cancer (CRC) is unclear. This association was examined by taking account of important confounding factors at the time of first presentation of symptoms in primary care.  **METHODS:** A total of 268 patients with CRC were included in a prospective, population-based study in a Danish county. The diagnostic interval was defined as the time from first presentation of symptoms until diagnosis. We analysed patients separately according to the general practitioner’s interpretation of symptoms. Logistic regression was used to estimate 3-year mortality odds ratios as a function of the diagnostic interval using restricted cubic splines and adjusting for tumour site, comorbidity, age, and sex.  **RESULTS:** In patients presenting with symptoms suggestive of cancer or any other serious illness, the risk of dying within 3 years decreased with diagnostic intervals up to 5 weeks and then increased (P=0.002). In patients presenting with vague symptoms, the association was reverse, although not statistically significant.  **CONCLUSION:** Detecting cancer in primary care is two sided: aimed at expediting ill patients while preventing healthy people from going to hospital. This likely explains the counterintuitive findings; but it does not explain the increasing mortality with longer diagnostic intervals. Thus, this study provides evidence for the hypothesis that the length of the diagnostic interval affects mortality in  CRC patients. | | | | | | | | | | |
| **376** | | **Thompson** | | **UK** | | **2010** | | **Colorectal Disease** | **3** | |
| *Is earlier referral and investigation of bowel cancer patients presenting with rectal bleeding associated with better survival?*  **Aim:** This study was carried out to determine whether rectal bleeding is related to stage of bowel cancer and whether earlier diagnosis and treatment are associated with improved survival.  **Method:** Eight hundred and forty-five patients were identified in the Wessex Bowel Cancer Audit (1991–1994). Presenting symptoms were identified from case notes. Outcome measures included 5-year survival, Dukes’ stage, metastatic disease at surgery and time from onset of symptoms to treatment, in patients presenting with rectal bleeding or other symptoms and signs.  **Results:** Six hundred and seventy-six (80%) of 845 patient case notes were reviewed. Of these, 408 (60.4%) patients had rectal or sigmoid cancer, and 255 (62.5%) of these 408 patients, who presented with rectal bleeding, had significantly earlier stage disease than those with a change in bowel habit and ⁄ or abdominal pain (Dukes’ stage A: 23.1% vs 3.6%; Dukes’ stage D: 14.5% vs 23.4%; P < 0.001), fewer metastases visible at surgery (14.9% vs 22.6%; P < 0.001) and significantly better 5-year survival (54.8% vs 40.9%; P < 0.001). There was no further significant improvement in 5-year survival in patients treated within 6 months of the onset of symptoms (55.1% vs 53.5%). Hazard ratios showed that 5-year survival was independently associated with age, Dukes’ stage and emergency treatment, but not with rectal bleeding, change in bowel habit, abdominal pain or delay in treatment.  **Conclusion:** Bowel cancer patients presenting with rectal bleeding had earlier stage disease and significantly better survival than patients presenting with a change in bowel habit or abdominal pain. There was no reduction in 5-year survival in those patients who had a delay in treatment for > 6 months from the onset of symptoms. | | | | | | | | | | |
